# Validity of automated audiometry for hearing examination in patients with multidrug-resistant tuberculosis

**DOI:** 10.12688/f1000research.75090.1

**Published:** 2021-12-14

**Authors:** Nyilo Purnami, Rian W. Palandeng, Soedarsono -, Dhany Arifianto, In Seok Moon

**Affiliations:** 1 Department of Otorhinolaryngology Head and Neck Surgery, Faculty of Medicine, Universitas Airlangga, Surabaya, Indonesia, Indonesia; 2 Department of Pulmonology and Respiratory Medicine, Faculty of Medicine, Universitas Airlangga, Surabaya, Indonesia, Indonesia; 3 Department of Engineering Physics, Institut Teknologi Sepuluh Nopember, Surabaya, Indonesia, Indonesia; 4 Department of Otorhinolaryngology, Yonsei University, Seoul, South Korea

**Keywords:** multidrug-resistant tuberculosis, ototoxicity, audiometry

## Abstract

**Background**: The objective of this study was to test the validity of automated audiometry as a method of hearing examination in patients with multidrug-resistant tuberculosis.

**Method** **s**: This was a cross-sectional comparative study with a retrospective approach, using patient medical records. Patients with multidrug-resistant tuberculosis (MDR-TB) were recruited based-on medical records that met the inclusion and exclusion criteria at the Pulmonology outpatient unit, then referred to the Otorhinolaryngology outpatient unit of the Dr. Soetomo Academic Medical Center. The subjects’ hearing function was measured with two different devices (automated audiometer and conventional audiometer) before being given anti-tuberculosis drug therapy (aminoglycoside injection) as ototoxicity monitoring from July to December 2019 period. Sensitivity and specificity analysis was used to assess the validity of the test.

**Results**: A total of 36 patients (72 ears) were included. The comparison test results using the Mann-Whitney test showed that there were significant differences between automated audiometry and conventional audiometry in both ears. Analysis values were: sensitivity 80-97%, specificity 37-96%, positive predictive value 74-98%, and negative predictive value 59-96%.

**Conclusion** **s**: Automated audiometry is valid for use as a method of hearing examination and monitoring in patients with multidrug-resistant tuberculosis.

## Introduction

Multidrug-resistant tuberculosis (MDR-TB) is tuberculosis (TB) resistant to isoniazid and rifampicin, with or without resistance to other anti-TB drugs. ^1^ The World Health Organization (WHO) recommendations for multidrug-resistant tuberculosis (MDR-TB) include 8+ months of an aminoglycoside treatment such as kanamycin or amikacin or capreomycin. Aminoglycosides can produce significant side effects, including irreversible ototoxicity. ^2^^,^^3^ The incidence of ototoxicity due to administration of aminoglycosides varies from 7% to 90%. Ototoxicity in MDR-TB patients is sensorineural and can be detected early by monitoring the hearing threshold periodically until the patient is recovered. Ototoxicity starts at high frequencies so that hearing techniques at high frequency are more sensitive to detect cochlear damage compared to methods that can only measure at standard frequencies (≤8000 Hz). ^4^^,^^5^

Initial hearing screening - at least air conduction (AC) - should be done on all patients who will start anti-tuberculosis drug therapy, especially aminoglycosides. Audiometry is a procedure to test one's listening ability at various sound frequencies and is used to identify hearing loss. This procedure is carried out using an electronic device called an audiometer to get the value of AC and bone conduction (BC). Not all audiometers can assess BC, so audiometry as auditory screening only requires AC values. MDR-TB patients with normal audiogram results can continue using anti-TB injections. ^6^^,^^7^

Conventional audiometry is a gold standard examination to assess hearing loss. This procedure is carried out in a soundproof booth to determine the hearing threshold, which is the lowest pure tone that someone can still hear at a specific frequency, from 250 to 8000 Hz. The audiometer consists of a sound intensity control knob, a frequency control knob, headphones to assess AC and BC. ^8^ Not all hospitals have soundproof chambers for this examination, and they are not recommended for MDR-TB patients because of the small size of the chamber. There is also less air circulation so they can cause shortness of breath and disturb concentration. ^6^^,^^7^

Automated audiometry is an audiometer device that, in its use, does not require a soundproof booth; or in other words, automated audiometry is a portable audiometer that can be used in an open space. There is an active noise monitoring feature that functions to monitor the high level of background noise when conducting audiometry, making it possible to pause the test until the background noise level returns to low. ^9^^,^^10^

Automated audiometry needs to be assessed for validity, and research in Indonesia has never been done. The purpose of this study was to prove the validity of automated audiometry as a method of hearing examination in patients with multidrug-resistant tuberculosis.

## Methods

This research was a cross-sectional comparative study with a retrospective approach. The subjects of this study were patients with MDR-TB in the Pulmonology outpatient unit Dr. Soetomo Academic Medical Center, who were referred to the otorhinolaryngology outpatient clinic for examination of hearing function, before starting the anti-tuberculosis drug therapy (aminoglycoside injection) as monitoring of ototoxicity during the period from July to December 2019. Data were retrieved from medical records that met the inclusion and exclusion criteria. Inclusion criteria were new MDR-TB patients who performed two kinds of hearing examination using conventional audiometry as the gold standard of hearing assessment and automated audiometry which can measure at high frequencies. Exclusion criteria were patients with incomplete medical record data.

Automated audiometry uses the KUDUwave audiometer (model KUDUwave Prime), which can measure at frequencies from 250 Hz to 16 000 Hz. The patient uses headphones in an open space of the Pulmonology outpatient department with a noise level of 60 dB and is asked to press a button when a tone is heard. Conventional audiometry uses the Interacoustics AD226 audiometer, which can measure at frequencies of 125 Hz to 8000 Hz. The patient uses headphones in a soundproof chamber at the Pulmonology outpatient department with a noise level of 28 dB, and asked to press a button when a tone is heard. Calibration of the two audiometers is done routinely. Examination with automated audiometry and conventional audiometry from medical record data in this study was carried out by competent medical personnel.

Data obtained from the medical records included air conduction (AC) results from conventional audiometry and automated audiometry examinations. Other data recorded included sex, age, results of an otoscopy examination, pure tone average (PTA), and the degree of hearing loss based on ear count. The automated audiometry examination results were compared with conventional audiometry results that were calculated at all frequencies. The subsequent analysis with IBM SPSS Statistics 25.0 uses a 2 × 2 table, with the output in sensitivity, specificity, positive predictive value (PPV), and negative predictive value (NPV). Comparative analysis of automated audiometry and conventional audiometry using the Mann Whitney test.

Ethical clearance was obtained from the Ethical Committee of Dr. Soetomo Hospital, Surabaya, Indonesia (approval number 1858/KEPK/111.2020). Written informed consent was obtained from all individuals included in this study, after being given an explanation of the examinations to be carried out.

## Results

### Basic data

Based on data from medical records, the results of hearing tests using two methods were compared: (i) automated audiometry examination conducted in the open field of a Pulmonology outpatient department, (ii) conventional audiometry performed in a soundproof room as the gold standard of hearing function examination. Data were obtained from 36 patients (72 ears) in the study period. There were 21 male patients (58.33%) and 15 female patients (41.67%). ^31^

The youngest MDR-TB patient was 18 years old, while the oldest was 85 years old. The largest age group was 45 to 54 years, with 13 patients (36.11%). The results of the otoscopy examination in 36 patients (72 ears) showed all normal tympanic membranes ( Table 1).

**Table 1.** Distribution of the respondents.

Age groups (years)	(n)	(%)
<15	0	0
15-24	4	11.11
25-34	4	11.11
35-44	10	27.78
45-54	13	36.11
55-64	4	11.11
≥65	1	2.78
<15	0	0
15-24	4	11.11
Total	36	100.00

Conventional audiometry examination obtained normal hearing with an average of 19.26 ± 4.42 dB, mild hearing loss with an average of 29.52 ± 3.39 dB, moderate with an average of 45.62 ± 3.92 dB, moderate to severe with 62.50 ± 4.68 dB, and severe hearing loss with an average of 81.25 ± 12.37 dB ( Table 2).

**Table 2.** Conventional audiometry. SD = standard deviation.

Degree of hearing loss (pure tone average [PTA])	Mean	Median	SD
Normal (≤25 dB)	19.26	20.00	4.42
Mild (26-40 dB)	29.52	28.75	3.39
Moderate (41-55 dB)	45.62	45.00	3.92
Moderate to severe (56-70 dB)	62.50	62.50	4.68
Severe (71-90 dB)	81.25	81.25	12.37
Profound (≥91 dB)	-	-	-

Automated audiometry examination results obtained normal hearing with an average of 16.93 ± 5.34 dB, mild hearing loss with an average of 31.67 ± 4.21 dB, moderate with an average of 50.78 ± 4.11 dB, moderate to severe degree with the average was 59.37 ± 0.88 dB and severe degree with an average of 87.50 ± 2.89 dB ( Table 3).

**Table 3.** Automated audiometry. SD = standard deviation.

Degree of hearing loss (pure tone average [PTA])	Mean	Median	SD
Normal (≤25 dB)	16.93	18.75	5.34
Mild (26-40 dB)	31.67	31.87	4.21
Moderate (41-55 dB)	50.78	51.87	4.11
Moderate to severe (56-70 dB)	59.37	59.37	0.88
Severe (71-90 dB)	87.50	87.50	2.89
Profound (≥91 dB)	-	-	-

The normality test results showed that the data were not normally distributed, so to find out significant differences between the two examinations, the Mann-Whitney test was used. The results showed significant differences (p < 0.05) between automated audiometry and conventional audiometry in both ears ( Table 4).

**Table 4.** Comparative automated audiometry with conventional audiometry.

	Pure tone average
Mann-Whitney U	2091.00
Wilcoxon W	4179.00
Z	−2.00
Sig. (2-tailed)	0.04

The automated audiometry test results compared with conventional audiometry results as the gold standard, obtained a sensitivity of 80-97%, specificity 37-96%, positive predictive value (PPV) 74-98%, and negative predictive value (NPV) 59-96% ( Table 5).

**Table 5.** Validity of automated audiometry. PPV = positive predictive value; NPV = negative predictive value.

	Degree of hearing loss					
	Normal	Mild	Moderate	Moderate to severe	Severe	Profound
Sensitivity	80%	89%	89%	97%	93%	-
Specificity	89%	37%	70%	85%	96%	-
PPV	95%	74%	86%	91%	98%	-
NPV	59%	64%	77%	96%	89%	-

## Discussion

The limitation of this study is that high frequencies (8000-16000 Hz) data collection of the automated audiometry was not carried out. The distribution of sex in this study found more male than female patients, consisting of 21 males (58.33%) and 15 females (41.67%). These results are consistent with research in China where 1154 MDR-TB incidents comprised 777 males and 377 females. ^11^ MDR-TB is more frequent in males, a fact that is supported by research in Rawalpindi, Pakistan, that reports MDR-TB is more dominant in males with 23 cases than in females with 15 cases. ^12^ However, a study in Ethiopia stated that the risk of MDR-TB decreases by 14% in males compared to females. ^13^

Another study in Surakarta reported MDR-TB cases in 50 males and 26 females. ^14^ The reason for this is not yet known, but could be due to male mobility or exposure due to social interactions is higher than female and non-compliance of a male patient in consuming anti-TB drugs. ^15^ A study about the risk of multidrug- or rifampicin-resistance in males versus females stated that there was no evidence of either sex being more at risk of MDR-TB. ^16^ The age characteristic of the youngest MDR-TB patients is 18 years, while the oldest is 85 years. The most populous age group was 45 to 54 years with 13 patients (36.11%). The average age of patients with MDR-TB was 43.44 years. Research in China reports that the most populous age group of MDR-TB patients is 31-45 years, with as many as 383 patients. ^11^ Other studies in Mali report as many as 134 of 214 MDR-TB patients, including in the age group ≤40 years. ^17^ A study in Gujarat reported that majority of MDR-TB patients were aged between 40 to 50 years. ^18^ Age groups between 24-50 were found more in this study, probably because of its higher activity than other age groups.

The comparison test results using the Mann-Whitney test showed significant differences between automated audiometry and conventional audiometry in both ears. Research on the accuracy and efficiency of automated audiometry reports that automated audiometry is a stable, accurate, and time-efficient method for evaluating adult hearing status with normal hearing and hearing loss. ^19^ Research in South Africa stated that there is no significant difference between conventional audiometry and automated audiometry. ^20^ Several reports included in a systematic review indicated that automated audiometry using the method of adjustment (Békésy sweep or Békésy fixed frequency method) generally yields lower (i.e., better) thresholds compared with manual audiometry. ^21^^–^^26^

Other studies report that conventional audiometry and audiometry hearing threshold results show a small difference. ^27^ Studies in primary school children aged 6-10 years in South Africa report that automated audiometry can correctly identify 87.5% of hearing loss detected using conventional audiometry. ^28^ Another study in industry reported that the difference in the hearing threshold between automated audiometry and conventional audiometry was less than 5 dB. ^29^ The difference in the results of the two examinations in this study was probably due to the difference in the frequency of the two devices used and the different conditions (fatigue, shortness of breath) of patients with MDR-TB when examined.

The automated audiometry results against the conventional audiometry results obtained 80-97% sensitivity, specificity 37-96%, positive predictive value 74-98%, and negative predictive value 59-96%. Research evaluating the sensitivity and specificity of automated audiometry reports that automated audiometry has a high sensitivity, ranging from 86-100% and specificity of 78-100%. Positive predictive value is around 89-91%, and negative predictive value is about 89-100%, indicating that automated audiometry can be used to identify hearing loss. ^30^ The results in this study were obtained according to the reference. The background noise level of a non-soundproofed room does not affect the accuracy of the hearing threshold value obtained using automated audiometry.

## Conclusions

This study shows that automated audiometry is a valid method of hearing examination and monitoring in patients with multidrug-resistant tuberculosis with normal hearing or hearing loss. Automated audiometry does not require a soundproof booth, rather can be performed in an open space. An active noise monitoring feature monitors the high level of background noise when conducting audiometry, making it possible to pause the test until the background noise level returns to low.

## Data availability

### Underlying data

Figshare: Validity of automated audiometry for hearing examination in patients with multidrug-resistant tuberculosis. https://doi.org/10.6084/m9.figshare.17129123. ^31^

Data are available under the terms of the Creative Commons Attribution 4.0 International license (CC-BY 4.0).
