# Supplementary material for: Validity of automated audiometry for hearing examination in patients with multidrug-resistant tuberculosis
Source: F1000Res. 2022 Aug 2;10:1277. Originally published 2021 Dec 14. [Version 2] doi: 10.12688/f1000research.75090.2 (PMC9468620; doi:10.12688/f1000research.75090.2)
Supplement: Peer review history

# Peer review: Reviewer response for version 2

**DOI:** 10.5256/f1000research.136850.r146234

**Published:** 2022-09-12

**Authors:** Jackie L. Clark

**Affiliations:** 1 Callier Center for Communication Disorders, University of Texas at Dallas, Dallas, TX, USA; 2 Department of Speech Pathology and Audiology, University of Witwatersrand, Johannesburg, South Africa

Thank you for thoughtfully reading through reviewer comments and providing your responses. 

 While I understand the authors found no clinically significant differences between the two conditions, I would strongly encourage the authors to add a comment in the conclusions of their MS that their findings demonstrated good noise attenuation by the equipment in moderately (less pristine) noisy environments, however it is important for all testers to be mindful of the manufacturer's warnings. 

 My concern is that there will be some readers that will interpret the study results encouraging test in any and every environment especially with exceedingly poor testing protocol within outrageously noisy (both auditorily and visually) environments. As a consequence, building the impression amongst other medical professionals that auditory measures are not reliable. Again, visual as well as auditory noise can be detrimental to all audiometric test results if the noise exceeds specific limits should also be noted by the authors. Otherwise, the authors are encouraging poor testing protocols and completely ignoring the manufacturer recommendations for using automated audiometry in an open environment.

 Thank you for responding with thoroughness of an adequate explanation. I DO believe it is important for the authors to conclude with very clear language that *for optimal use they* *encourage all testing sites to follow the manufacturer's suggested guidelines for use of equipment*. 

 I completely understand that in this situation, the researchers were aware of the limits of acceptable and optimal environmental. It is also important as leaders in research to encourage others to conduct proper and ethical testing. Unfortunately, our uninformed colleagues may not be aware of these important issues of maintaining proper and ethical test environments.

Is the work clearly and accurately presented and does it cite the current literature?

Yes

If applicable, is the statistical analysis and its interpretation appropriate?

Yes

Are all the source data underlying the results available to ensure full reproducibility?

Yes

Is the study design appropriate and is the work technically sound?

Partly

Are the conclusions drawn adequately supported by the results?

Partly

Are sufficient details of methods and analysis provided to allow replication by others?

Yes

Reviewer Expertise:

NA

I confirm that I have read this submission and believe that I have an appropriate level of expertise to confirm that it is of an acceptable scientific standard.

# Peer review: Reviewer response for version 1

**DOI:** 10.5256/f1000research.78907.r123563

**Published:** 2022-04-13

**Authors:** Jackie L. Clark

**Affiliations:** 1 Callier Center for Communication Disorders, University of Texas at Dallas, Dallas, TX, USA; 2 Department of Speech Pathology and Audiology, University of Witwatersrand, Johannesburg, South Africa

Though there are a number of studies over the past number of years across the globe with similar findings. I only have a few minor concerns: the authors indicated that there were significant threshold differences between the automated vs conventional audiometry; and conclusion suggests that the automated audiometry is a valid method of hearing examination. The significant differences may be statistically different, and though they don't appear with the limited data available, there is no clinically significant difference between the two conditions.

 The other concern is that the automated audiometry was completed in a relatively hostile environment. The manufacturer suggests that while a sound booth is not necessary, it IS necessary to find an auditorily and visually quiet space. If the system were used appropriately (i.e. in an environment without visual and auditory competition) the findings may have revealed less difference between the two conditions. It is important to keep in mind that the advantage of the automated audiometry is the capability of task shifting to an assistant so that the audiologist can work on more advanced measures in the clinic. The noise suppression system within the automated system optimizes the assessment outcomes.

 Thank you for the opportunity of reviewing this interesting manuscript.

Is the work clearly and accurately presented and does it cite the current literature?

Yes

If applicable, is the statistical analysis and its interpretation appropriate?

Yes

Are all the source data underlying the results available to ensure full reproducibility?

Yes

Is the study design appropriate and is the work technically sound?

Partly

Are the conclusions drawn adequately supported by the results?

Partly

Are sufficient details of methods and analysis provided to allow replication by others?

Yes

Reviewer Expertise:

Teleaudiology, pediatric audiology, electrophysiology, large scale community based hearing screenings, hearing aid fitting and dispensing.

I confirm that I have read this submission and believe that I have an appropriate level of expertise to confirm that it is of an acceptable scientific standard, however I have significant reservations, as outlined above.

# Response

**Published:** 2022-07-26

**Authors:** Nyilo Purnami

**Affiliations:** PERHATI-THTKL, Indonesia

1. *"The authors indicated that there were significant threshold differences between the automated vs conventional audiometry; and conclusion suggests that the automated audiometry is a valid method of hearing examination."*
**I Agree, in here the authors want to test the validity of automated auditory as method hearing examination because there are already differences in the regulation of the examination, namely without the use of canamicyn injection. it is hoped that automated auditory can be used**
2. *"The significant differences may be statistically different, and though they don't appear with the limited data available, there is no clinically significant difference between the two conditions"*
**Because the research is limited in time, we using patient medical records in a six month period. There is no clinically significant different because patient and equipment limitations.**
3. *"The manufacturer suggests that while a sound booth is not necessary, it IS necessary to find an auditorily and visually quiet space."*
**An active noise monitoring feature monitors the high level of background noise when conducting audiometry, making it possible to pause the test unlit the background noise level. But thank you for suggestions, may be the next research we will consider carefully about manufacturer suggest.**
4. *"If the system were used appropriately (i.e. in an environment without visual and auditory competition) the findings may have revealed less difference between the two conditions. It is important to keep in mind that the advantage of the automated audiometry is the capability of task shifting to an assistant so that the audiologist can work on more advanced measures in the clinic. The noise suppression system within the automated system optimizes the assessment outcomes."*
**​​​​​​​The advantage of this examination is that it provides benefits and convenience to an assistant so that the audiologist can work on more advanced measures in the clinic.**

# Peer review: Reviewer response for version 1

**DOI:** 10.5256/f1000research.78907.r115235

**Published:** 2022-02-02

**Authors:** Herman A. Jenkins

**Affiliations:** 1 Department of Otolaryngology, University of Colorado School of Medicine, Aurora, CO, USA

Access and therefore expense of providing standard audiometry testing is often difficult, particularly in developing countries with areas of limited access. This manuscript compares standard audiometry with an automated form. The latter does not require sound dampened facilities or great expense. The investigators compared the two and demonstrated that, while the automated testing was less predictive of hearing level, it was within a range to make it feasible for situations not allowing sophisticated setup.

 The authors have used a small, but valid sample and the conclusions that they have drawn are valid. Monitoring in conditions as drug resistance or chemotherapy is important and the use of automated testing provided sufficient information to validate the hearing level without requiring expensive set up. I would recommend indexing the manuscript as is.

Is the work clearly and accurately presented and does it cite the current literature?

Yes

If applicable, is the statistical analysis and its interpretation appropriate?

Yes

Are all the source data underlying the results available to ensure full reproducibility?

Yes

Is the study design appropriate and is the work technically sound?

Yes

Are the conclusions drawn adequately supported by the results?

Yes

Are sufficient details of methods and analysis provided to allow replication by others?

Yes

Reviewer Expertise:

I am Chair of an Otolaryngology-Head and Neck Surgery Department in the USA with expertise in hearing and balance disorders and monitoring both.

I confirm that I have read this submission and believe that I have an appropriate level of expertise to confirm that it is of an acceptable scientific standard.

# Response

**Published:** 2022-02-03

**Authors:** Nyilo Purnami

**Affiliations:** PERHATI-THTKL, Indonesia

Thank you very much.
